# Complete Genome Sequence of Rotavirus Group C Isolated in South Korea

**DOI:** 10.1128/genomeA.01068-15

**Published:** 2015-09-24

**Authors:** Dong-Uk Kim, Kwi-Sung Park, Jae-Kyung Kim, Shien-Young Kang, Kyung-Ah Yoon

**Affiliations:** a Chungcheongnam-Do Health and Environment Research Institute, Daejeon, South Korea; b College of Veterinary Medicine, Chungbuk National University, Cheongju, South Korea; c Department of Biomedical Laboratory Science, College of Health Sciences, Dankook University, Cheonan, South Korea; d Department of Clinical Pathology, Daejeon Health Sciences College, Daejeon, South Korea

## Abstract

Rotavirus group C is the major etiological agent associated with acute gastroenteritis in all human age groups. Here, we report the complete genome sequence of human group C rotavirus (GpC-RV) isolated in South Korea.

## GENOME ANNOUNCEMENT

Rotaviruses (RVs) belong to the family *Reoviridae* and are major viral pathogens causing acute gastroenteritis in humans and animals worldwide (1). The RV genome contains 11 segments of double-stranded RNA (dsRNA) enclosed within triple concentric layers and is categorized into seven groups (A to G) according to serological and genomic properties of VP6 (2). Group C rotavirus (GpC-RV) strain Cowden was first recognized in swine in 1980 (3). Since then, GpC-RV has been identified in humans, cattle, ferrets, and dogs (4–7). Infections of human GpC-RV associated with sporadic cases or large outbreaks of acute diarrhea in all age groups have been reported around the world, although most of these infections in humans are from group A rotavirus (GpA-RV) (8–10). However, some serological studies indicated that positive rates of antibodies for human GpC-RV were 3 to 45% in the human population in certain geographic areas (11–13). Furthermore, recent investigations have found evidence for transmission from swine to humans and gene reassortment between human GpC-RVs (14, 15). These studies suggest that GpC-RV may be an emerging human pathogen. Nevertheless, the complete genome sequences of human GpC-RVs that have been reported so far are very limited worldwide. It is therefore necessary to analyze the complete genome of human GpC-RV for molecular epidemiology studies.

In this study, human GpC-RV strain Chungnam was isolated from a stool sample from a 4-year-old female with acute gastroenteritis who had been hospitalized at the department of pediatrics at Dankook University Hospital in Cheonan, South Korea, in February 2014, and its complete sequences were determined for the 11 dsRNA segments.

Viral RNA was extracted from the supernatant of the fecal samples using an automated MagNA Pure instrument (Roche Applied Science), according to the manufacturer’s instructions. cDNA synthesis was performed using a reverse transcription system (Promega) with a random hexamer primer. Eleven complete genes from overlapping fragments were amplified by PCR using 34 primer pairs described by Yamamoto et al. (15) designed based on strain YNR001 (GenBank accession no. HQ185652 to HQ185662). Overlapping amplicons purified were sequenced directly on an automated ABI 3730 sequencer (Applied Biosystems) and assembled using MegAlign (DNAStar) and Clustal W (version 1.81). The complete genome sequence of Chungnam was 17,910 nucleotides (nt) long. The sizes of the 11 genomes (VP7, VP4, VP6, VP1, VP2, VP3, NSP1, NSP2, NSP3, NSP4, and NSP5) from Chungnam were 1,063, 2,283, 1,353, 3,309, 2,736, 2,166, 1,270, 1,037, 1,350, 613, and 730 nt, respectively.

Phylogenetic analysis indicated that complete nucleotide sequences of Chungnam showed the highest similarity (99.5%) with strain YNR001 clustered in the China-Japan branch, and 10 genes were closely related to YNR001, except for the NSP5 gene, which shared the highest homology with strain BK0830 (99.4%). These results suggest that Chungnam may be a predominant human GpC-RV strain worldwide, considering that it was also clustered in the same branch with recent strains isolated from Hungary and China (16).

### Nucleotide sequence accession numbers.

The nucleotide sequences for Chungnam determined in this study have been deposited in the GenBank database under accession numbers KM886899 to KM886901 for VP7, VP4, and VP6, respectively, and KP844855 to KP844862 for NSP1 to -5 and VP1 to -3, respectively.
